# Sertoli cell tumor associated with ovarian sex cord tumor with annular tubules in a patient with 46 XY disorder of sex development and 9p24.3 deletion, case report

**DOI:** 10.1111/jog.70027

**Published:** 2025-08-01

**Authors:** Tabatha Petrillo, Christian Battipaglia, Elisa Semprini, Cinzia Baldessari, Marta Pirola, Olga Calabrese, Antonino Farulla, Giliana Ternelli, Albino Eccher, Laura Botticelli, Alessandro D. Genazzani

**Affiliations:** ^1^ Division of Obstetrics and Gynaecology AUSL of Modena Carpi Italy; ^2^ Department of Medical and Surgical Sciences for Mother, Child and Adult University of Modena and Reggio Emilia, Azienda Ospedaliero Universitaria Policlinico Modena Italy; ^3^ Clinical and Experimental Medicine PhD Programme, Department of Biomedical, Metabolic and Neural Sciences University of Modena and Reggio Emilia Modena Italy; ^4^ Department of Oncology and Hematology Azienda Ospedaliero‐Universitaria Policlinico Modena Italy; ^5^ Medical Genetic Unit University Hospital of Modena Modena Italy; ^6^ Department of Pathology, Policlinico di Modena University of Modena and Reggio Emilia Modena Italy

**Keywords:** 9p24.3 deletion, disorder of sex development, primary amenorrhea, Sertoli cell tumor, sex cord tumor with annular tubules

## Abstract

We report a rare case involving a 22‐year‐old phenotypically female patient who presented to our care with primary amenorrhea and spontaneous breast development. Hormonal analysis indicated hypergonadotropic hypogonadism, and imaging revealed a hypoplastic uterus and calcified ovaries. Karyotyping was 46, XY and the presence of the *SRY* gene was confirmed. The patient underwent laparoscopic bilateral salpingo‐oophorectomy due to the high risk of malignancy development. Histopathological analysis revealed bilateral Sertoli cell tumors and a sex cord tumor with annular tubules in the right gonad. Next generation sequencing genetic testing identified a 1.24 Mb deletion on chromosome 9p24.3, which included the DMRT1, DMRT2, and DMRT3 genes, as well as a partial deletion of KANK1. Hormonal replacement therapy was not initiated due to the potential risk of tumor recurrence, and follow‐up imaging was scheduled every 6 months for the first 2 years and then annually. No recurrence was observed at 24 months.

## INTRODUCTION

Disorders of sexual development (DSD) are a heterogeneous group of congenital conditions characterized by discordance between chromosomal, gonadal, and phenotypic sex.^1^ They are classified as 46, XY DSD, 46, XX DSD, or sex chromosome DSDs.^2^

Among 46 XY DSDs, the second by frequency is complete gonadal dysgenesis (CGD), also called Swyer Syndrome, with an incidence of 1 in 80 000 births.^2^ A Y chromosome in dysgenetic gonads is associated with a high risk for neoplastic transformation into germ cell tumors (GCTs), especially gonadoblastoma and dysgerminoma.^1^

Sex cord tumor with annular tubules (SCTAT) is a rare ovarian neoplasm with features intermediate between granulosa and Sertoli cell tumors and accounts for <1% of ovarian sex cord tumors.^3^

We present, to our knowledge, the first reported case of a phenotypically female 46,XY patient with bilateral Sertoli cell tumors and SCTAT.

## CASE REPORT

A 22‐year‐old woman was referred to our clinic for primary amenorrhea. She had not undergone any hormonal assessment, but her gynecologist had prescribed a combined estrogen‐progestin contraceptive pill for 3 years, which resulted in regular withdrawal bleeding. She stopped using it 6 months prior to our evaluation, and no menstruation had occurred since. Written informed consent was obtained from the patient to collect and publish clinical and pathological data.

**FIGURE 1:**
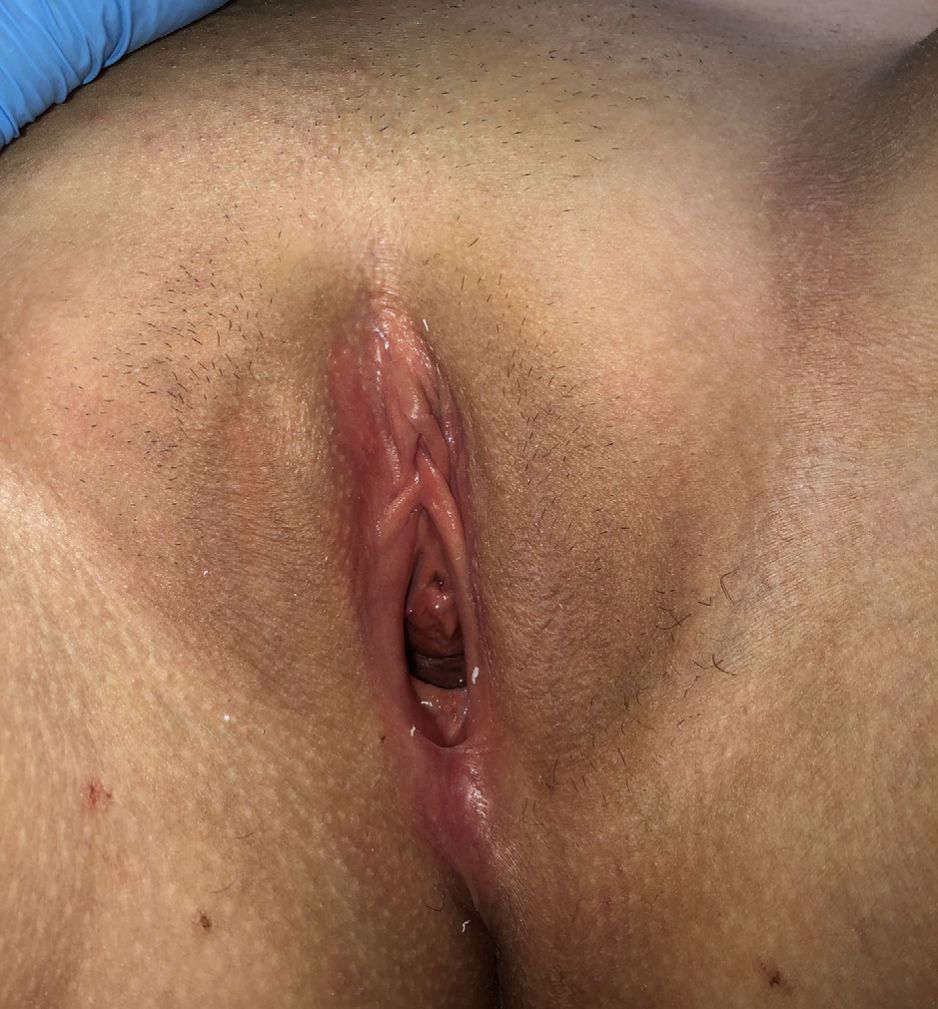
External genitalia at clinical examination showed a normal female appearance without signs of virilization or ambiguity. Hypoestrogenic features were present.

On examination, her height was 176 cm, and her weight was 95 kg. She showed Tanner stage B5 breast development and P3 pubic hair. External genitalia appeared normal, with no signs of virilization, although hypoestrogenic features were noted (Figure 1).

**FIGURE 2:**
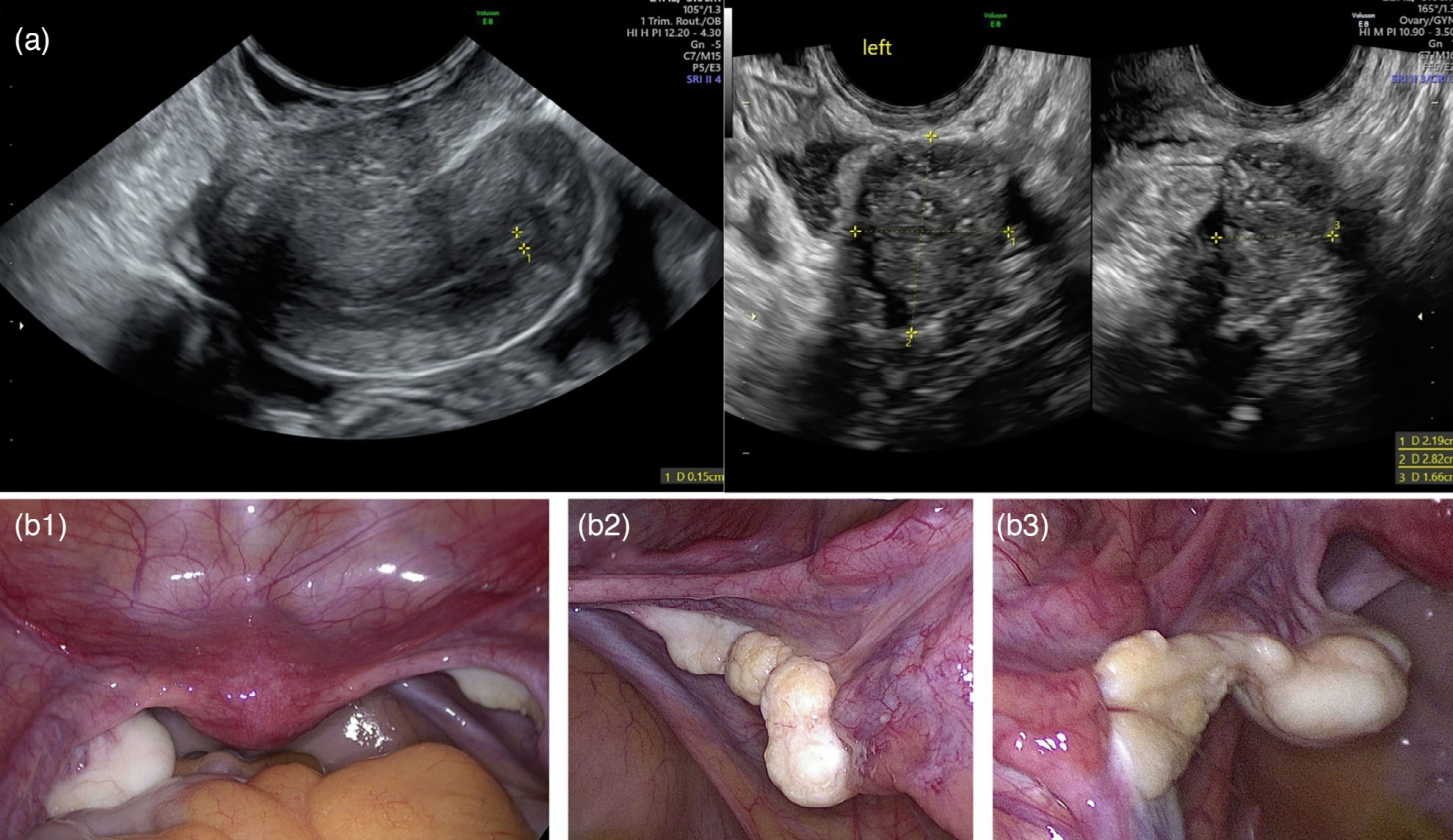
(a) Pelvic ultrasound showing a hypoplastic uterus measuring 42 × 18 × 29 mm with thin endometrium (on the left) and the left adnexa revealing a nonfunctional ovary with hyperechoic foci suggestive of calcifications (on the right). (b) 1. Laparoscopic view of the uterus and adnexa; 2. right adnexa; 3. left adnexa.

A pelvic ultrasound revealed a hypoplastic uterus, bilateral ovaries without functional signs, and calcifications in the left ovary (Figure 2a).

After 6 months hormonal therapy discontinuation, laboratory evaluation revealed follicle stimulating hormone 93.9 mIU/mL, luteinizing hormone 34.3 mIU/mL, estradiol less than 19 pg/mL, progesterone 0.4 ng/mL, prolactin 10.7 ng/mL, thyroid stimulating hormone 7.40 mcIU/mL, fT4 8.5 pg/mL, fT3 4.3 pg/mL, testosterone 0.4 ng/mL, anti‐Müllerian hormone (AMH) less than 0.1 ng/mL. Results indicated hypergonadotropic hypogonadism and subclinical hypothyroidism.

Karyotype analysis revealed a 46,XY profile, with the SRY gene confirmed by fluorescence in situ hybridization FISH (Yp11.3). Genetic counseling excluded consanguinity or familial disorders. Given the female phenotype, presence of a uterus, and Y chromosome, CGD was suspected. Next generation sequencing (NGS) of DSD‐related genes was requested.

The genetic counseling for DSD is a comprehensive, sensitive, and individualized process that helps patients and families understand the biological, medical, and emotional aspects of these complex conditions. The process is usually structured in two sessions: a pre‐test session during which clinical observations and the rationale for genetic testing are explained and a post‐test session, where the results are communicated in a supportive setting. In both sessions, psychological support is proposed. The patient is also offered the option to be accompanied by parents or other trusted individuals to favor social and emotional support at the time of diagnosis.

In our patient's case, particular attention was given to the emotional impact of a 46,XY diagnosis in the context of a female gender identity. Given her young age, the key elements of reproductive potential and long‐term hormonal care were first discussed in a one‐on‐one session with the patient, followed by a second session with her mother present to support shared understanding.

To find malformations associated with CGD and to detect and stage possible tumors, an abdominal computed tomography (CT) and evaluation of tumor markers were performed. At the CT scan, the uterus was small, both ovaries showed calcifications, and no alterations attributable to the substitutive nature were described. Moreover, serum β human chorionic gonadotropin, lactate dehydrogenase, and alpha fetoprotein were within the normal range.

According to recommendations from the Consensus statement on management of intersex disorders,^4^ the patient underwent laparoscopic bilateral salpingo‐oophorectomy and peritoneal washing^2^ (Figure 2b).

**FIGURE 3:**
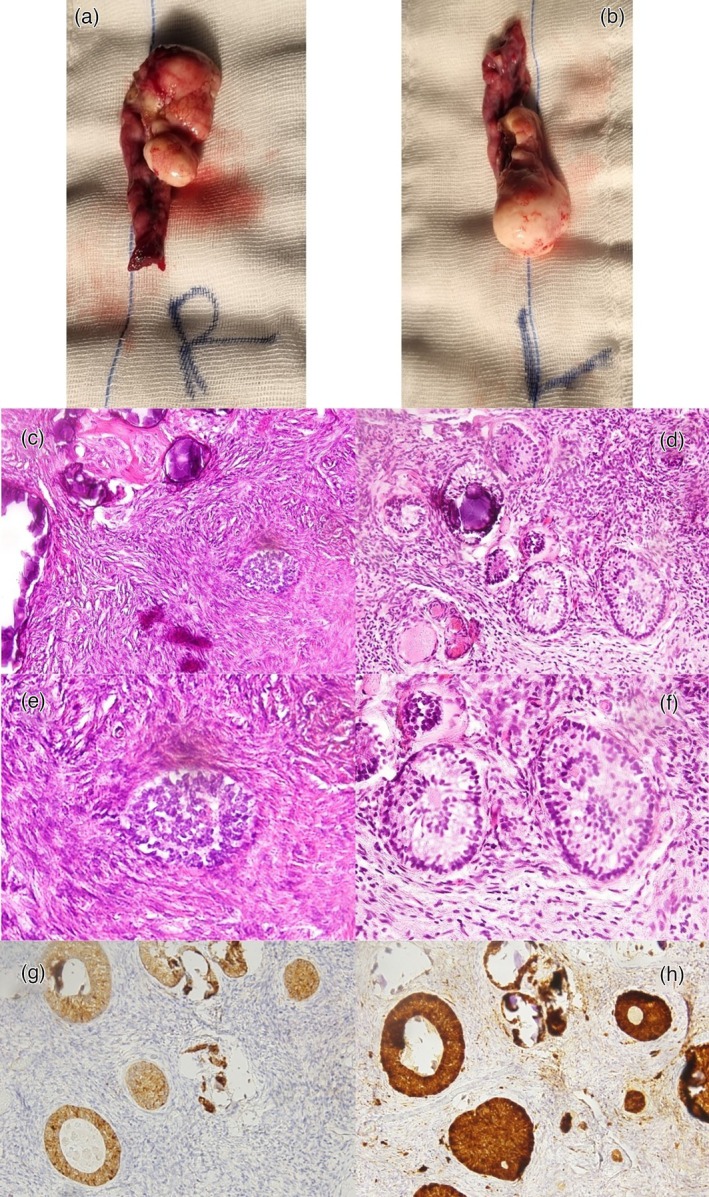
Macroscopic aspect of (a) right adnexa and (b) left adnexa; histopathological and immunohistochemical features of the right gonad. (c) Hematoxylin and eosin (H&E) stain displaying fibromatous stroma with calcifications and focal nests of tubular structures; (d) H&E stain highlighting well‐defined annular tubules, characteristic of sex cord tumor with annular tubules (SCTAT); (e) 40× microscopic enlargement showing features of Sertoli cell tumor; (f) 40× microscopic enlargement highlighting ring‐shaped tubules of SCTAT; (g) immunohistochemical staining for inhibin showing cytoplasmic positivity in sex cord‐stromal cells; (h) Calretinin immunostaining with diffuse strong positivity in tumor cells, supporting the diagnosis of a sex cord‐stromal tumor.

The peritoneal washing revealed no signs of neoplastic cells. Pathologic macroscopy reported a left ovary of 5.5 × 2.5 × 1.5 cm with a lumpy neoformation and a solid grayish surface; the right ovary measured 4 × 2 × 1 cm and was described as irregular‐surfaced and grayish (Figure 3a,b).

Microscopically, the left gonad was composed of ovarian parenchyma with a fibroma showing calcification and bony metaplasia. The right ovarian parenchyma lacked follicles and showed calcifications, a fibroma with bony metaplasia, and small tubular nests composed of tall cells with pale cytoplasm and basal nuclei. Immunohistochemistry showed positive staining for calretinin, WT1, and inhibin, and negative staining for EMA and CD10. These elements were evocative of a SCTATs (Figure 3c–h).

Due to the complexity and rarity of the findings, histopathological findings were submitted for a second opinion to another reference center for oncological diseases. There, histological slide review showed immunohistochemistry positive for Calretinin, inhibin, and CA125 (only in the superficial epithelium) while negative for SALL4. In the right gonad, an ovary‐type internal tissue without follicles was confirmed; the tumor was reported as a Sertoli cell tumor, calcifying type, in an abundant fibromatous stroma, with a modest component of SCTAT. In the left ovary, a sclerosing fibroma with multiple calcifications and a component of calcifying‐type Sertoli cell tumor was also observed.

Given the low stage and absence of high‐risk features, follow‐up with pelvic/abdominal ultrasound or CT every 6 months for 2 years, then annually, was recommended. Hormone replacement therapy was deferred due to the potential risk of tumor recurrence.^5^

Over 1 year later, NGS revealed a complete deletion of the DMRT1 gene. A subsequent CGH array disclosed a 1.24‐Mb deletion in the 9p24.3 chromosome, including DMRT1, DMRT2, DMRT3, and partially KANK1 gene (exons 2 and 12).

At 24‐month follow‐up, no signs of disease recurrence were observed.

## DISCUSSION

CGD is a rare form of 46, XY DSD characterized by the presence of typical Müllerian structures, absent androgenization, and non‐functional streak gonads. It typically presents in adolescence with primary amenorrhea and absent breast development due to hypoestrogenism.^2^

The SRY gene on the Y chromosome initiates the differentiation of the undifferentiated gonad into a testis. SRY activates the expression of several genes, such as SOX9 and FGF9, while repressing Wnt4, overall inducing male gonad differentiation.^6^ The secretion of AMH from Sertoli cells causes regression of the Müllerian ducts, while testosterone secreted from Leydig cells promotes the differentiation of the Wolffian ducts into seminal vesicles, vas deferens, and epididymis. In the absence of a Y chromosome, the Müllerian structures persist and form fallopian tubes, uterus, and upper third of the vagina.^2^, ^7^

Approximately 15% of all cases of CGD result from a mutation involving the SRY gene. Mutations in NR5A1, DMRT1, and MAP3K1 are other leading causes, but together with mutations in the SRY gene, they explain less than 40% of all non‐syndromic forms of 46 XY gonadal dysgenesis.^1^

Our patient was 22 years old, with female external genitalia, showed primary amenorrhea, but had spontaneous breast development at Tanner's stage B5. In literature, other cases of spontaneous breast development and menstruation have been reported.^7^ Breast development is usually believed to be induced by the hormonal activity of tumors within the dysgenetic gonad, estrogen production by streak gonads, peripheral aromatization of androgens, or enhanced estrogen sensitivity.^8^, ^9^

The presence of a 46XY karyotype, hypergonadotropic hypogonadism, Müllerian structures, female phenotype, and the absence of virilization led us to diagnose and treat the patient as having Swyer Syndrome rather than other conditions such as complete androgen insensitivity syndrome, Mayer–Rokitansky–Küster–Hauser syndrome, and congenital adrenogenital hyperplasia (CAH).^1^

Genetic analysis later revealed a 1.24 Mb deletion at 9p24.3 that involved the DMRT1, DMRT2, DMRT3, and KANK1 genes (exons 2 and 12), which allowed the clinical picture to be classified among the 9p‐deletion syndromes.

The 9p deletion syndrome (9PMS), also named “Alfi syndrome” (OMIM #158170), is characterized by craniofacial anomalies, intellectual disability, and other congenital defects.^10^ However, 9p24.3 deletions have been observed in patients with 46, XY sex reversal without other features of the 9PMS, leading to the definition of 46, XY gonadal dysgenesis, partial or complete, with 9p24.3 deletion (OMIM #154230).

Following the genetic diagnosis, the patient underwent cardiological assessments, electroencephalography, neurological and orthopedic evaluations, and an abdominal and thoracic CT scan, all excluding abnormalities typically associated with 9PMS.

Bilateral gonadectomy is recommended for the high risk of developing GCTs when the diagnosis of 46 XY DSD is made.^1^ While dysgerminoma and gonadoblastoma are more common, our patient had bilateral Sertoli cell tumors with a right‐sided component of SCTAT.^2^, ^11^

Sertoli cell tumors are rare ovarian sex cord‐stromal tumors, often with a non‐aggressive clinical course, and typically diagnosed at stage I. They may secrete estrogens and, rarely, androgens or progesterone.^11^ The primary treatment is unilateral oophorectomy, while lymphadenectomy should only be performed in cases of suspicious nodes upon imaging or intraoperative examination. For patients of reproductive age, fertility‐sparing surgery that preserves the uterus and one ovary is safe in the early stages of the disease. Ovarian sex‐cord stromal tumors are characterized by an indolent course and late recurrence (median time to relapse of 4–6 years). Therefore, long‐term follow‐up is recommended.^11^

SCTAT is an even rarer tumor with an incidence of <1% among the ovarian sex cord tumors. It exhibits histologic features between granulosa and Sertoli cell tumors. They are usually associated with Peutz–Jeghers syndrome (PJS), but our case was not syndromic. Non‐PJS SCTATs are typically unilateral, unifocal, larger, and have demonstrated malignant potential, including lymph node metastasis and recurrence.^12^

Several rare case reports have highlighted the association between SCTAT and DSD. Moon et al. reported a 24‐year‐old woman with Turner syndrome and SCTAT, indicating a possible connection between dysgenetic gonads and gonadoblastoma‐like histology.^13^ Similarly, Shi et al. documented SCTAT in a 16‐year‐old with true hermaphroditism,^14^ while Young et al. described a 28‐year‐old woman with a normal karyotype who had bilateral dysgerminomas, a unilateral gonadoblastoma, and SCTAT.^15^ These cases support the hypothesis by Hart et al. that SCTAT may be a neoplastic transformation of the sex cord component of gonadoblastoma, especially in dysgenetic or abnormal gonadal environments.^16^ Although SCTAT is typically linked to PJS, its presence in non‐syndromic patients with 46,XY DSD, as in our case, further supports the wider range of tumorigenic pathways involved in abnormal gonadal development.

The most frequent clinical manifestations of SCTAT are related to hyperestrinism, such as menstrual irregularities, postmenopausal bleeding, and precocious puberty.^12^ Our patient presented with low levels of estradiol at the time of diagnosis, although levels at tumor onset are unknown.

There is no standardized treatment for SCTAT due to its rarity. The initial management of patients with SCTAT is surgery. Ovarian cystectomy is not recommended due to the high recurrence risk.^17^ Unilateral salpingo‐oophorectomy is feasible for patients with an intact capsule and tumor confined to one ovary. Biopsy of the contralateral ovary should be considered. Ipsilateral pelvic and para‐aortic lymphadenectomy may be considered for suspected metastases, though its routine role is unclear.^18^ Chemotherapy is reserved for advanced or recurrent cases.^18^

The aims of hormone replacement therapy (HRT) in DSD patients are to induce and sustain sexual development, along with other aspects of puberty, including growth.^4^ It helps to induce and maintain adequate breast development, menstruation, and optimal peak bone mass.^1^ HRT also has the potential to prevent morbidity and excess mortality correlated with premature menopause.^15^ Despite the lack of female gonads, pregnancy in CGD is possible via oocyte donation and in vitro fertilization, as these patients have typical Müllerian structures.^7^

Despite all the beneficial effects, HRT use in the presence of ovarian cancer remains controversial. Current guidelines indicate that HRT therapy is allowed after non‐epithelial ovarian tumors, excluding granulosa cell tumors.^5^ Due to the possibility of granulosa‐like differentiation in SCTATs, HRT was not recommended for our patient.

## CONCLUSION

We described a rare case of CGD with 9p24.3 deletion in a 46,XY phenotypically female patient, presenting with bilateral Sertoli cell tumors and a component of SCTAT. This association, to the best of our knowledge, has never been reported before.

This case highlights the importance of considering DSD in the diagnostic work‐up of primary amenorrhea, even in the presence of spontaneous puberty. Karyotype analysis and comprehensive endocrine and genetic evaluation are essential for timely diagnosis and appropriate management.

## AUTHOR CONTRIBUTIONS

**Tabatha Petrillo:** Writing – original draft; investigation; conceptualization. **Christian Battipaglia:** Conceptualization; investigation; writing – review and editing. **Elisa Semprini:** Supervision; validation. **Cinzia Baldessari:** Validation. **Marta Pirola:** Visualization. **Olga Calabrese:** Validation. **Antonino Farulla:** Visualization. **Giliana Ternelli:** Visualization. **Albino Eccher:** Visualization. **Laura Botticelli:** Validation. **Alessandro D. Genazzani:** Project administration; supervision.

## CONFLICT OF INTEREST STATEMENT

The authors declare no conflict of interests for this article.

## Data Availability

Data sharing is not applicable to this article as no new data were created or analyzed in this study.

## References

[1] Wisniewski AB , Batista RL , Costa EMF , Finlayson C , Sircili MHP , Dénes FT , et al. Management of 46,XY differences/disorders of sex development (DSD) throughout life. Endocr Rev. 2019;40(6):1547–1572. 31365064 10.1210/er.2019-00049

[2] King TFJ , Conway GS . Swyer syndrome. Curr Opin Endocrinol Diabetes Obes. 2014;21(6):504–510. 25314337 10.1097/MED.0000000000000113

[3] Yahaya JJ , Mshana D , Mremi A . Ovarian sex cord tumour with annular tubules in a 13‐year‐old female: a case report. Oxf Med Case Rep. 2020;2020(4):omaa024. 10.1093/omcr/omaa024 PMC7243713 32477574

[4] Lee PA , Houk CP , Ahmed SF , Hughes IA . International consensus conference on intersex organized by the Lawson Wilkins Pediatric Endocrine Society and the European Society for Paediatric Endocrinology. Consensus statement on management of intersex disorders. International consensus conference on intersex. Pediatrics. 2006;118(2):e488–e500. 16882788 10.1542/peds.2006-0738

[5] Rousset‐Jablonski C , Selle F , Adda‐Herzog E , Planchamp F , Selleret L , Pomel C , et al. Fertility preservation, contraception and menopause hormone therapy in women treated for rare ovarian tumours: guidelines from the French national network dedicated to rare gynaecological cancers. Eur J Cancer. 2019;116:35–44. 31170563 10.1016/j.ejca.2019.04.018

[6] Capel B . Vertebrate sex determination: evolutionary plasticity of a fundamental switch. Nat Rev Genet. 2017;18(11):675–689. 28804140 10.1038/nrg.2017.60

[7] Morawiecka‐Pietrzak M , Dąbrowska E , Gliwińska A , Góra A , Geisler G , Gawlik A , et al. A rare case of primary amenorrhoea and breast development in a 46,XY 15‐year‐old girl. Pediatr Endocrinol Diabetes Metab. 2021;27(1):62–67. 33599439 10.5114/pedm.2020.101803 PMC10227485

[8] Fukamatsu Y , Tsukahara Y , Hayashi S , Yoshikawa F , Fukuta T . Bilateral gonadoblastoma producing steroid hormones in a patient with 45,X/46,XY gonadal dysgenesis. Gynecol Obstet Invest. 1990;30(3):189–191. 1702401 10.1159/000293266

[9] Tanwani LK , Chudgar D , Murphree SS , Eblen AC , Mokshagundam SPL . A case of gonadal dysgenesis, breast development, graves' disease, and low bone mass. Endocr Pract. 2003;9(3):220–224. 12917064 10.4158/EP.9.3.220

[10] Starosta RT , Jensen N , Couteranis S , Slaugh R , Easterlin D , Tate V , et al. Using a new analytic approach for genotyping and phenotyping chromosome 9p deletion syndrome. Eur J Hum Genet. 2024;32(9):1095–1105. 38972963 10.1038/s41431-024-01667-y PMC11369271

[11] Ray‐Coquard I , Morice P , Lorusso D , Prat J , Oaknin A , Pautier P , et al. Non‐epithelial ovarian cancer: ESMO clinical practice guidelines for diagnosis, treatment and follow‐up. Ann Oncol. 2018;29(suppl 4):iv1–iv18. 29697741 10.1093/annonc/mdy001

[12] Scully RE . The prolonged gestation, birth, and early life of the sex cord tumor with annular tubules and how it joined a syndrome. Int J Surg Pathol. 2000;8(3):233–238. 11493995 10.1177/106689690000800312

[13] Moon WS , Lee DG . Ovarian sex cord tumor with annular tubules in a patient with Turner syndrome. J Korean Med Sci. 1998;13(1):89–94. 9539327 10.3346/jkms.1998.13.1.89 PMC3054339

[14] Shi S , Tang M , Li W , Wu H , Liu Y , Luo Y , et al. True hermaphroditism with sex cord tumor with annular tubules (SCTAT): a rare case report and review of the literature. BMC Womens Health. 2022;22(1):551. 36575516 10.1186/s12905-022-02137-7 PMC9793495

[15] Young KMN , Scurry J , Jaaback K , Bowden NA , Scott RJ . Bilateral dysgerminoma associated with gonadoblastoma and sex‐cord stromal tumour with annular tubules in a 28‐year‐old fertile woman with normal karyotype. Pathology (Phila). 2012;44(3):257–260. 10.1097/PAT.0b013e32835140a5 22437743

[16] Hart WR , Kumar N , Crissman JD . Ovarian neoplasms resembling sex cord tumors with annular tubules. Cancer. 1980;45(9):2352–2363. 7379031 10.1002/1097-0142(19800501)45:9<2352::aid-cncr2820450920>3.0.co;2-#

[17] Lele SM , Sawh RN , Zaharopoulos P , Adesokan A , Smith M , Linhart JM , et al. Malignant ovarian sex cord tumor with annular tubules in a patient with Peutz‐Jeghers syndrome: a case report. Mod Pathol. 2000;13(4):466–470. 10786816 10.1038/modpathol.3880079

[18] Qian Q , You Y , Yang J , Cao D , Zhu Z , Wu M , et al. Management and prognosis of patients with ovarian sex cord tumor with annular tubules: a retrospective study. BMC Cancer. 2015;15:270. 25886261 10.1186/s12885-015-1277-y PMC4408581

